# Maternal Dietary Betaine Prevents High-Fat Diet-Induced Metabolic Disorders and Gut Microbiota Alterations in Mouse Dams and Offspring From Young to Adult

**DOI:** 10.3389/fmicb.2022.809642

**Published:** 2022-04-05

**Authors:** Jieying Liu, Lu Ding, Xiao Zhai, Dongmei Wang, Cheng Xiao, Xiangyi Hui, Tianshu Sun, Miao Yu, Qian Zhang, Ming Li, Xinhua Xiao

**Affiliations:** ^1^Key Laboratory of Endocrinology, Ministry of Health, Department of Endocrinology, Peking Union Medical College Hospital, Peking Union Medical College, Chinese Academy of Medical Sciences, Beijing, China; ^2^Department of Medical Research Center, Peking Union Medical College Hospital, Peking Union Medical College, Chinese Academy of Medical Sciences, Beijing, China

**Keywords:** gut microbiota, betaine, glucose and lipid metabolism, high-fat diet, dams and offspring, intergeneration

## Abstract

Early life is a critical window for preventing the intergenerational transmission of metabolic diseases. Betaine has been proven to play a role in improving glucose and lipid metabolism disorders in animal models. However, whether maternal betaine supplementation plays a role in regulating gut microbiota in both dams and offspring remains unclear. In this study, C57BL/6 female mice were fed with control diet (Ctr), high-fat diet (HF), and high-fat with betaine supplementation (0.3% betaine in the diet, HFB) from 3 weeks prior to mating and lasted throughout pregnancy and lactation. After weaning, the offspring got free access to normal chow diet until 20 weeks of age. We found that maternal dietary betaine supplementation significantly improved glucose and insulin resistance, as well as reduced free fatty acid (FFA) concentration in dams and offspring from young to adult. When compared to the HF group, *Intestinimonas* and *Acetatifactor* were reduced by betaine supplementation in dams; *Desulfovibrio* was reduced in 4-week-old offspring of the HFB group; and *Lachnoclostridium* was enriched in 20-week-old offspring of the HFB group. Moreover, the persistent elevated genus *Romboutsia* in both dams and offspring in the HFB group was reported for the first time. Overall, maternal betaine could dramatically alleviate the detrimental effects of maternal overnutrition on metabolism in both dams and offspring. The persistent alterations in gut microbiota might play critical roles in uncovering the intergenerational metabolic benefits of maternal betaine, which highlights evidence for combating generational metabolic diseases.

## Introduction

Metabolic disorders, including obesity, insulin resistance, hyperlipidemia, and glucose intolerance, have been rapidly increasing in prevalence globally and have become a major threat to the world (Harris et al., 1998; Kahn and Flier, 2000). Observations and experimental approaches have generally demonstrated that phenotypic outcomes with long-term consequences were involved in the interplay between genetic and environmental influences (Vishvanath and Gupta, 2019). The concept of developmental origins of health and disease (DOHaD) theory has been widely accepted, which proposed that early life environmental factors have profound effects on vulnerability to disease in adulthood (Haugen et al., 2015). Extensive range of clinical and epidemiological data suggested that gestational obesity and diabetes leave lasting impacts on the next generation, and so, the cycle of risk is perpetuated (Godfrey et al., 2017; Chen et al., 2021b; Du et al., 2021a; Jiang et al., 2021; Lorenzon et al., 2021). Experimental research in animal models also showed that maternal high-fat diet (HF) during pregnancy and lactation predisposed offspring to metabolic disorders (Zheng et al., 2014; Ribaroff et al., 2017). To reduce the burden of chronic disease in the next generation, management of maternal nutrient supplementation during pregnancy has been discovered to improve perinatal and offspring outcomes (Mathews et al., 2001; Cano-Ibanez et al., 2020).

Much of the literature demonstrated that the intake of bioactive dietary supplementations during pregnancy, including multivitamins (Yoshida et al., 2020), folic acid (Kulkarni et al., 2011), and other micro-nutrients, might prevent metabolic disorders in offspring. Betaine is commonly found in daily food like wheat, shellfish, and *Lycium barbarum* (Craig, 2004; Ejaz et al., 2016; Chen et al., 2021a). The beneficial effects of betaine on metabolic disorders, such as obesity and diabetes, have been extensively studied (Ejaz et al., 2016; Chen et al., 2021a). A number of clinical studies have confirmed that, compared with healthy people, the blood concentration of betaine was much lower in type 2 diabetes mellitus (T2DM) patients (Lever et al., 2012, 2014). In terms of pregnancy metabolic disorders, women who subsequently developed gestational diabetes mellitus (GDM) exhibited lower plasma betaine (Diaz et al., 2011). A clinical trial was conducted in an Asian mother–offspring cohort to examine the effects of maternal betaine status in neonatal birth weight and adiposity (van Lee et al., 2016). The authors found that higher maternal betaine status was associated with smaller infant birth weight and less abdominal fat mass (van Lee et al., 2016). Meanwhile, animal studies have also discovered that maternal dietary betaine supplementation could modify hepatic metabolism in newborn piglets. The underlying mechanism might be the epigenetic modulation of DNA methylation in IGF2/H19 (Yang et al., 2018), CYP7A1 (Zhao et al., 2019), HMGCR (Cai et al., 2014), and AMPK/LXR-mediated pathway (Cai et al., 2016).

The gastrointestinal tract is one of the largest interfaces linking the outside world and the internal environment in humans and animals. Gut microbiota, colonized in the intestinal tract through generations, play important roles in host health (Moeller et al., 2018). Obesity and glucose and lipid metabolism are generally associated with gut microbial dysbiosis, with evidence showing significant differences in the gut microbiota between metabolic disorder and healthy individuals (Craig, 2004; Ejaz et al., 2016). Dietary betaine also elevated *Akkermansia muciniphila*, which produce short-chain fatty acids (SCFAs; Du et al., 2021b). SCFAs were associated with the regulation of miR-378a family, which is involved in lipolysis and thermogenesis. Therefore, Betaine might exert its metabolic benefits *via* the gut microbiota-derived epigenetic modulation regulatory axis (Du et al., 2021b). However, whether maternal betaine supplementation plays a role in influencing gut microbiota in both dams and offspring remains unclear.

In this study, we explored the metabolic beneficial effect of maternal betaine supplementation prior to mating, during pregnancy, and lactation on preventing glucose and lipid metabolic disorders in dams and offspring from young to adult. Moreover, we investigated the maternal gut microbiota alteration and whether these microbiome ecology changes could transmit to the next generation consistently.

## Materials and Methods

### Animal Study

C57BL/6 female mice, purchased from the Beijing HFK Bioscience Co., Ltd. (Beijing, China, SCXK-2016-0006), were maintained in a specific pathogen-free (SPF) environment (22 ± 2°C with 12-h light/dark cycle) with *ad libitum* access to food and water. After 1-week adaptation, 6-week-old mice with similar body weight were randomly assigned into three groups: the control group (Ctr, *n* = 24), which were fed a standard rodent diet (AIN-93G) (15.8% of the calories as fat); high-fat group (HF, *n* = 24), which were fed a high-fat diet (D12492) (60% of the calories as fat); and high-fat with betaine group (HFB, *n* = 24), which were fed a high-fat diet and 0.3% betaine (purity ≥99%, Sigma) in the diet. After the 3-week intervention, C57BL/6 female mice were mated with 9-week-old males, which was confirmed by visualization of a vaginal plug. All the female mice received their original diets during pregnancy and lactation. At birth, the litter sizes were culled to five pups to ensure that there is no nutritional bias between litters. Pups were weaned at 21 days of age. Subsequently, they received a standard chow diet until 20 weeks of age. Body weights of dams and offspring were measured every week. All the works were performed according to procedures approved by the Animal Care and Ethics Committee at Peking Union Medical College Hospital (Beijing, China, XHDW-2019-012). All the animal operations were conducted in compliance with the National Institutes of Health guide for the care and use of laboratory animals.

### Glucose- and Insulin-Tolerance Tests

For glucose tolerance test, mice that fasted for 14–16 h were orally administered a glucose load (2 g/kg of body weight). For insulin tolerance test, mice that fasted for 6 h were injected intraperitoneally with rapid-acting insulin (1.0 U/kg of bodyweight). Blood glucose levels were monitored from tail bleeding before intervention and 15, 30, 60, 90, and 120 min after intervention using a glucometer (FreeStyle Optium™, Abbott). The area under the curve (AUC) was calculated as previously described (Zheng et al., 2014).

### Plasma Biochemical Analyses

Blood samples were collected from the intraorbital retrobulbar plexus from mice after 16 h of fasting, separated by centrifugation at 3,000 × *g* for 10 min at 4°C, and the plasma was stored at −80°C. Plasma glucose, free fatty acids (FFAs), total triglyceride (TG), total cholesterol (TC), high-density lipoprotein cholesterol (HDL-C), low-density lipoprotein cholesterol (LDL-C), alanine transaminase (ALT), and aspartate aminotransferase (AST) were measured as previously described (Zhou et al., 2019b).

### Enzyme-Linked Immunosorbent Assay

Plasma insulin was detected using the enzyme-linked immunosorbent assay (ELISA) kit (ELR-Insulin-1, RayBiotech Life, Inc., Atlanta, United States), according to the protocol provided by the supplier. Insulin sensitivity was assessed by the homeostasis model assessment of insulin resistance (HOMA-IR), which was calculated as previously described (Zheng et al., 2014).

### Fecal Microbiota Analysis

Bacterial genomic DNA was extracted from the cecal contents using CTAB method. The V3–V4 regions of the 16S rRNA genes were amplified using the primers 341F 5′-CCTAYGGG RBGCASCAG-3′ and 806R 5′-GGACTACNNGGGTATCTAAT-3′ with the barcode. Mixture PCR products was purified with the Qiagen Gel Extraction Kit (Qiagen, Dusseldorf, Germany) and quantified using the Qubit^®^ 2.0 Fluorometer (Thermo Fisher Scientific, New York, United States). The tags were sequenced on the Illumina NovaSeq platform. Raw tags were quality-filtered under specific filtering conditions to obtain high-quality clean tags (Bokulich et al., 2013) according to QIIME (version 1.9.1)^1^ quality-controlled process (Caporaso et al., 2010). Operational taxonomic units (OTUs) were clustered with a 97% similarity cutoff using UPARSE (version 7.0.1001)^2^ (Edgar, 2013). The Silva Database^3^ was used to annotate representative sequences based on Mothur algorithm (Quast et al., 2013). To detect α-diversity, species diversity indices including Shannon and Simpson indexes were performed. To visually evaluate β-diversity, Bray–Curtis principal coordinate analysis (PCoA) was performed. The discriminated bacterial taxa among groups were identified with linear discriminant analysis effect size (LEfSe) analysis (Segata et al., 2011).

### Statistical Analyses

All data were presented as mean ± standard error of the mean (S.E.M). Statistics were analyzed by one-way ANOVA and two-way ANOVA, with Tukey’s *post hoc* analyses. A *p*-value < 0.05 was considered statistically significant. Correlation analyses between the relative abundance of bacterial taxa at genus levels and metabolic parameters were performed by Spearman’s correlation coefficient test. Prism version 8.0 (GraphPad Software Inc., San Diego, CA, United States) was used for statistical analysis.

## Results

### Betaine Improves Glucose and Lipid Metabolism in High-Fat Diet Dams

**FIGURE 1:**
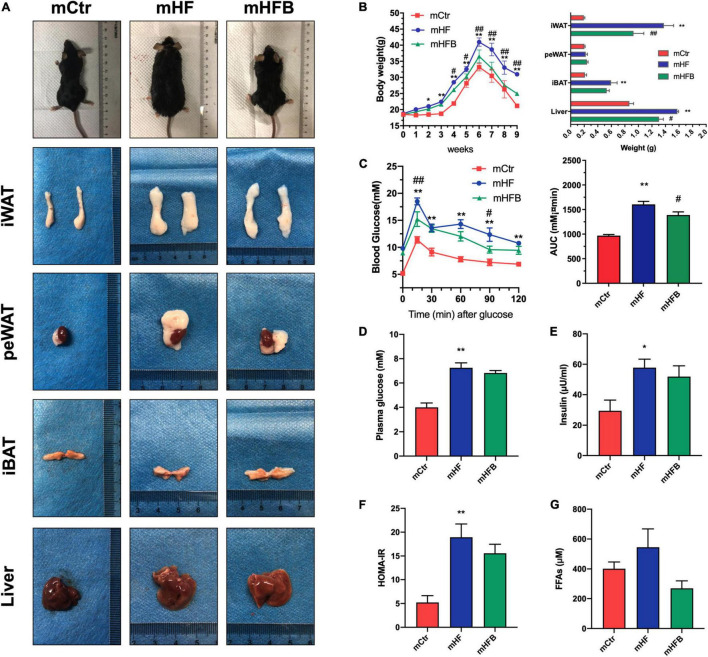
Betaine improves glucose and lipid metabolism in high-fat diet dams. Dams were analyzed after weaning. **(A)** General picture of body composition; **(B)** body weight (left) and body composition (right) changes during the betaine treatment; **(C)** oral glucose tolerance test (left) and the area under the curve (right); **(D)** fasting plasma glucose; **(E)** fasting insulin level; **(F)** HOMA-IR; **(G)** FFAs. Ctr, standard control diet; HF, high-fat diet; HFB, high-fat diet with betaine; HOMA-IR, homeostasis model assessment of insulin resistance; FFAs, free fatty acids. Data are expressed as means ± S.E.M. (*n* = 4–6/group). One-way and two-way ANOVA; *p < 0.05 and **p < 0.01 vs. Ctr, #*p* < 0.05 and ##*p* < 0.01 vs. HF.

In order to investigate the metabolic benefits of betaine in dams, female mice were dietary supplemented with betaine for 3 weeks prior to mating and during pregnancy and lactation. As demonstrated in Figures 1A,B, HF feeding led to a significant increase in body weight of dams when compared to the control group (*p* < 0.05), which was significantly reduced from the third week after betaine supplementation (*p* < 0.05). As for the body composition of dams, betaine dramatically decreased the subcutaneous white adipose tissue (sWAT) and liver weight elevation by HF diet (*p* < 0.05) and tended to reduce the weight of brown adipose tissue (BAT). However, no significant change was found regarding the weight of perirenal white adipose tissue (peWAT). Oral glucose tolerance test (OGTT) was performed to detect the glucose metabolism of mice (Figure 1C). HF led to a remarkable higher blood glucose (*p* < 0.01) and larger AUC values (*p* < 0.01) compared to control diet, and maternal betaine feeding significantly reduced blood glucose levels at 15 min (*p* < 0.01) and 90 min (*p* < 0.01), as well as AUC values (*p* < 0.05). Fasting glucose and insulin were also detected. Both glucose (*p* < 0.01) and insulin level (*p* < 0.05) were significantly elevated by HF, while betaine contributed to a trend toward lower glucose and insulin levels (Figures 1D,E). Meanwhile, betaine treatment also tended to reduce the HOMA-IR index in dams *per se*, which was induced by HF (Figure 1F). To assess the lipid metabolism, plasma biochemical indexes were detected. Maternal betaine significantly reduced the level of TC (*p* < 0.05) (Supplementary Figure 1A) and tended to reduce the levels of FFAs in dams compared to the HF group (*p* = 0.07) (Figure 1G). There was no observable difference in the levels of TG, LDL-C, and HDL-C between the HF and HFB groups (Supplementary Figures 1B–D). ALT and AST were tested to evaluate liver injury. Both ALT and AST were decreased after betaine feeding compared to the HF group (*p* < 0.05) (Supplementary Figures 1E,F).

### Betaine Alters the Gut Microbiota in High-Fat Diet Dams

**FIGURE 2:**
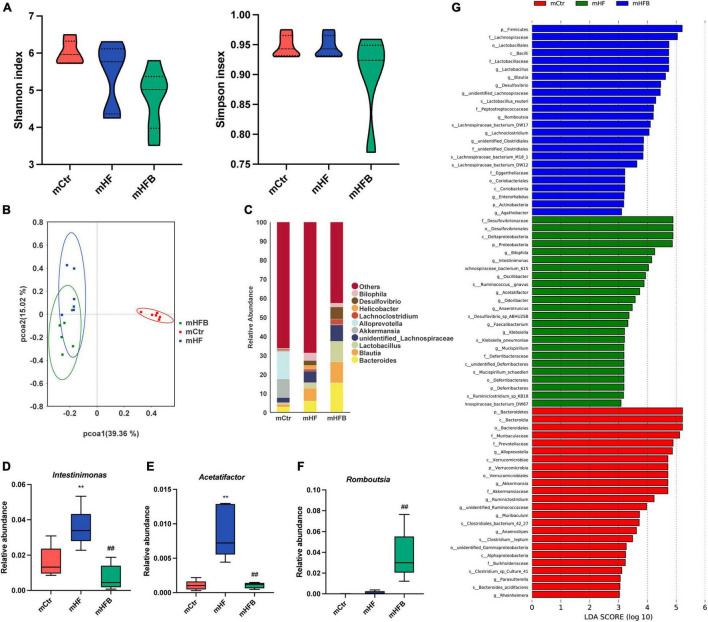
Betaine alters the gut microbiota in high-fat diet dams. The cecal contents were collected from dams after weaning. **(A)** Shannon index (left) and Simpson index (right); **(B)** PCoA plots of gut microbiome; **(C)** relative abundance of the top 10 species at the genus level; **(D)** relative abundance of *Intestinimonas*; **(E)** relative abundance of *Acetatifactor*; **(F)** relative abundance of *Romboutsia*; **(G)** distinct taxa identified in the three groups using LEfSe analysis. The colors represent the group in which the indicated taxa is more abundant compared to the other group. Ctr, standard control diet; HF, high-fat diet; HFB, high-fat diet with betaine. Data are expressed as means ± S.E.M. (*n* = 4–6/group). One-way ANOVA; **p < 0.01 vs. Ctr, ##*p* < 0.01 vs. HF.

The 16S rRNA gene sequencing was applied to investigate the impact of maternal betaine treatment on gut microbiota in dams. The α-diversity was evaluated by species diversity indices including Shannon and Simpson indexes. The result showed that microbial diversity had no difference among the three groups (Figure 2A). A β-diversity analysis was performed to explore whether HF or betaine treatment was associated with altered microbial composition. As shown in Figure 2B, PCoA revealed that the intestinal microbiota of dams was separate among the three groups, which was further confirmed by ANOSIM (*R* = 0.3148, *p* = 0.003 comparing mHFB with mHF, data not shown). The relative abundance of the top 10 genera is listed in Figure 2C. At the genus level, maternal HF significantly increased the relative abundance of *Intestinimonas* and *Acetatifactor* (*p* < 0.01) when compared to the control group, which was markedly reduced by betaine supplementation (*p* < 0.01) (Figures 2D,E). Intriguingly, the relative abundance of *Romboutsia* was dramatically elevated by betaine feeding compared to HF feeding in dams (*p* < 0.01) (Figure 2F). LEfSe analysis showed the enriched abundance from the phylum to the genus level. We found that *Blautia* was enriched after betaine administration in dams (Figure 2G).

### Maternal Betaine Ameliorates Glucose and Lipid Metabolism Disorders Induced by Early-Life High-Fat Diet in 4-Week Offspring

**FIGURE 3:**
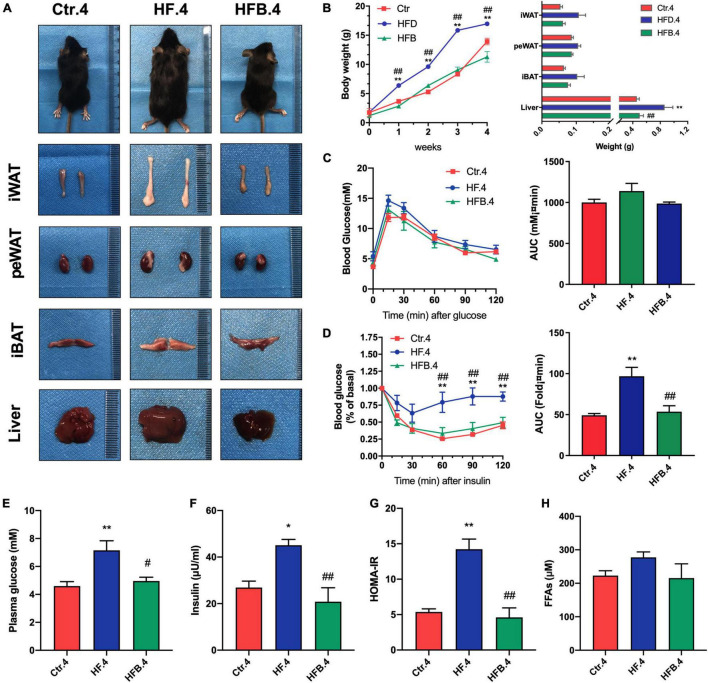
Maternal betaine ameliorates glucose and lipid metabolism induced by early-life high-fat diet in 4-week-old offspring. Offspring mice were analyzed at 4 weeks of age. **(A)** General picture of body composition; **(B)** body weight (left) and body composition (right) changes; **(C)** oral glucose tolerance test (left) and the area under the curve (right); **(D)** insulin tolerance test (left) and the area under the curve (right); **(E)** fasting plasma glucose; **(F)** fasting insulin level; **(G)** HOMA-IR; **(H)** FFAs. Ctr.4, 4-week-old offspring of dams fed with the standard control diet; HF.4, 4-week-old offspring of dams fed with the high-fat diet; HFB.4, 4-week-old offspring of dams fed with the high-fat diet with betaine. HOMA-IR, homeostasis model assessment of insulin resistance; FFAs, free fatty acids. Data are expressed as means ± S.E.M. (*n* = 4–6/group). One-way and two-way ANOVA; *p < 0.05 and **p < 0.01 vs. Ctr, #*p* < 0.05 and ##*p* < 0.01 vs. HF.

Offspring received a standard chow diet after weaning until 20 weeks of age. To investigate the intergenerational effects of maternal betaine supplementation in young offspring, an investigation was conducted on the glucose and lipid metabolism in 4-week-old mice. In line with dams, maternal betaine feeding significantly reduced body weight (*p* < 0.01) and liver weight (*p* < 0.05), which were elevated by HF. Besides, betaine tended to reduce the weight of adipose tissues (Figures 3A,B). OGTT showed no difference among the three groups, but maternal betaine led to a smaller AUC in 4-week-old offspring (Figure 3C). In terms of insulin sensitivity, insulin tolerance test (ITT) showed that maternal HF dramatically elevated the blood glucose at 60, 90, and 120 min (all *p* < 0.01) and led to a larger AUC (*p* < 0.01) (Figure 3D). By contrast, maternal betaine significantly reduced the blood glucose at 60, 90, and 120 min (all *p* < 0.01) and led to a smaller AUC (*p* < 0.01), when compared with the HF group (Figure 3D). The results showed that maternal betaine contributed to improve the insulin sensitivity of offspring at 4 weeks of age. Besides, maternal HF also dramatically elevated the fasting glucose (*p* < 0.01), fasting insulin (*p* < 0.05), and HOMA-IR (*p* < 0.01), whereas maternal betaine played a role in reducing the level of fasting glucose (*p* < 0.05), fasting insulin (*p* < 0.01), and HOMA-IR (*p* < 0.01) compared to the HF group (Figures 3E–G). Moreover, FFAs showed a trend of decrease after maternal betaine treatment (Figure 3H). However, there was no significant difference in TG, TC, LDL-C, HDL-C, ALT, and AST among the three groups (Supplementary Figures 2A–F).

### Maternal Betaine Alters the Gut Microbiota in Early-Life High-Fat Diet Offspring at 4 Weeks of Age

**FIGURE 4:**
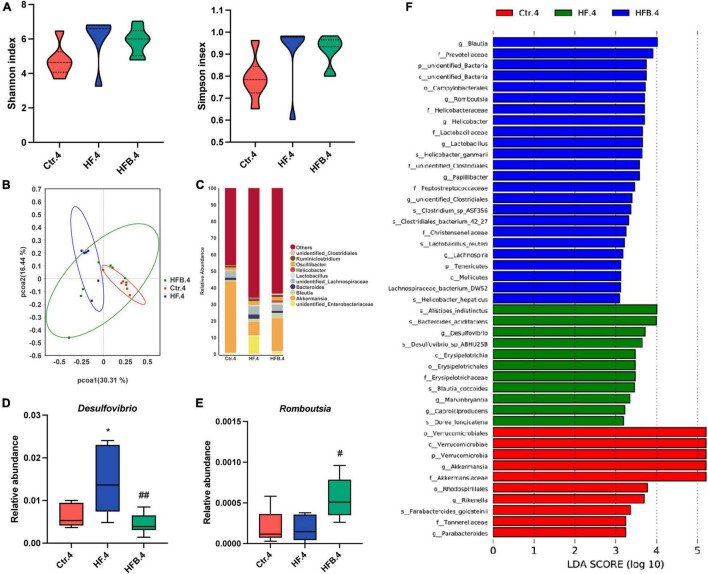
Maternal betaine alters the gut microbiota in early-life high-fat diet offspring at 4 weeks of age. The cecal contents were collected from offspring at 4 weeks of age. **(A)** Shannon index (left) and Simpson index (right); **(B)** PCoA plots of gut microbiome; **(C)** relative abundance of the top 10 species at the genus level; **(D)** relative abundance of *Desulfovibrio*; **(E)** relative abundance of *Romboutsia*; **(F)** distinct taxa identified in the three groups using LEfSe analysis. The colors represent the group in which the indicated taxa is more abundant compared to the other group. Ctr.4, 4-week-old offspring of dams fed with the standard control diet; HF.4, 4-week-old offspring of dams fed with the high-fat diet; HFB.4, 4-week-old offspring of dams fed with the high-fat diet with betaine. Data are expressed as means ± S.E.M. (*n* = 6–8/group). One-way ANOVA; *p < 0.05 vs. Ctr, #*p* < 0.05 vs. HF.

We also explored the influence of maternal betaine on the microbiota in young offspring at 4 weeks of age. The results showed that the α-diversity (Shannon and Simpson indexes) had no difference among the three groups (Figure 4A). PCoA demonstrated that the gut microbiota was separated among the three groups in 4-week-old offspring (Figure 4B). Figure 4C shows the relative abundance of the top 10 genera. At the genus level, maternal HF significantly improved the relative abundance of *Desulfovibrio* (*p* < 0.05), which was markedly reduced by maternal betaine (*p* < 0.01) (Figure 4D). Consistent with the dams, the relative abundance of *Romboutsia* was dramatically elevated by maternal betaine feeding compared to HF feeding (*p* < 0.05) (Figure 4E). Simultaneously, *Blautia* was also found enriched after maternal betaine administration in young offspring by LEfSe analysis (Figure 4F), which was consistent with the dams.

### Maternal Betaine Protects 20-Week-Old Offspring From Glucose and Lipid Metabolic Disorders Induced by Early-Life High-Fat Diet

**FIGURE 5:**
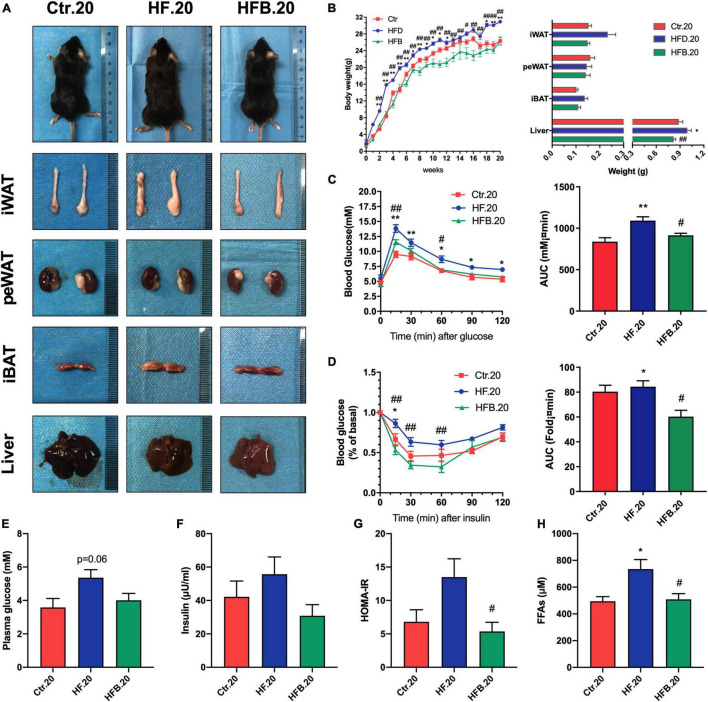
Maternal betaine protects 20-week-old offspring from glucose and lipid metabolic disorders induced by early-life high-fat diet. Offspring mice were analyzed at 20 weeks of age. **(A)** General picture of body composition; **(B)** body weight (left) and body composition (right) changes; **(C)** oral glucose tolerance test (left) and the area under the curve (right); **(D)** insulin tolerance test (left) and the area under the curve (right); **(E)** fasting plasma glucose; **(F)** fasting insulin level; **(G)** HOMA-IR; **(H)** FFAs. Ctr.20, 20-week-old offspring of dams fed with the standard control diet; HF.20, 20-week-old offspring of dams fed with the high-fat diet; HFB.20, 20-week-old offspring of dams fed with the high-fat diet with betaine. HOMA-IR, homeostasis model assessment of insulin resistance; FFAs, free fatty acids. Data are expressed as means ± S.E.M. (*n* = 5–8/group). One-way and two-way ANOVA; **p < 0.01 vs. Ctr, #*p* < 0.05 vs. HF.

Whether the effects of maternal betaine supplementation in glucose and lipid metabolic disorders were persistent from young to adult remains obscure; thus, further investigation was performed in offspring at 20 weeks of age. Figures 5A,B shows that maternal betaine markedly led to reduced body weight and liver weight elevated by HF feeding (*p* < 0.01), and maternal betaine also tended to reduce the weight of sWAT and BAT, whereas no significant difference was found in peWAT. As for glucose metabolism, maternal HF significantly elevated the blood glucose from 15 min after the oral administration of glucose (all *p* < 0.05) and led to a larger AUC (*p* < 0.01) (Figure 5C). Conversely, maternal betaine feeding dramatically reduced the blood glucose at 15 (*p* < 0.01) and 60 min (*p* < 0.05), as well as contributed to a smaller AUC compared to HF (*p* < 0.05) (Figure 5C). ITT showed that maternal HF significantly elevated the blood glucose at 15 min (*p* < 0.05) and led to a larger AUC (*p* < 0.05), whereas maternal betaine feeding markedly reduced the blood glucose at 15 (*p* < 0.01), 30 (*p* < 0.01), and 60 min (*p* < 0.01) and contributed to a smaller AUC compared to HF (*p* < 0.05) (Figure 5D). Meanwhile, maternal betaine tended to reduce the plasma fasting glucose and insulin (Figures 5E,F). Figure 5G reveals that maternal betaine significantly reduced the HOMA-IR index, suggesting that maternal betaine improved the insulin sensitivity of offspring at 20 weeks of age. Maternal betaine also played a role in reducing FFAs, which was elevated by maternal HF (*p* < 0.05) (Figure 5H). Additionally, maternal betaine reduced the TC level (*p* < 0.05), while no significant difference was found in TG, LDL-C, HDL-C, ALT, and AST level in adult offspring at 20 weeks of age (Supplementary Figures 3A–F).

### Maternal Betaine Alters the Gut Microbiota in Early-Life High-Fat Diet Offspring at 20 Weeks of Age

**FIGURE 6:**
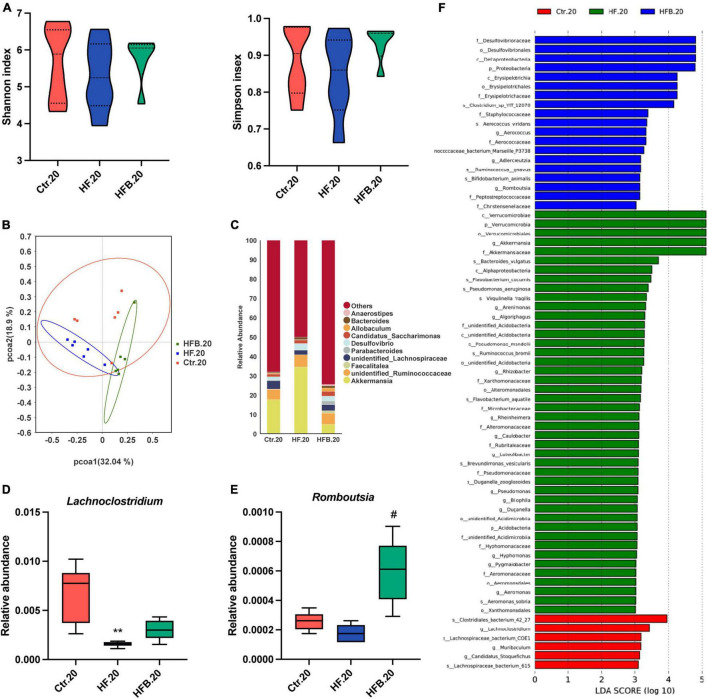
Maternal betaine alters the gut microbiota in early-life high-fat diet offspring at 20 weeks of age. The cecal contents were collected from offspring at 20 weeks of age. **(A)** Shannon index (left) and Simpson index (right); **(B)** PCoA plots of gut microbiome; **(C)** relative abundance of the top 10 species at the genus level; **(D)** relative abundance of *Desulfovibrio*; **(E)** relative abundance of *Romboutsia*; **(F)** distinct taxa identified in the three groups using LEfSe analysis. The colors represent the group in which the indicated taxa is more abundant compared to the other group. Ctr.20, 20-week-old offspring of dams fed with the standard control diet; HF.20, 20-week-old offspring of dams fed with the high-fat diet; HFB.20, 20-week-old offspring of dams fed with the high-fat diet with betaine. Data are expressed as means ± S.E.M. (*n* = 5–6/group). One-way ANOVA; **p < 0.01 vs. Ctr, #*p* < 0.05 vs. HF.

Considering the persistent protective effects of maternal betaine on offspring metabolism from young to adult, we further explored the alteration of gut microbiota in offspring at 20 weeks of age. Consistently, Shannon and Simpson indexes were similar among the three groups, suggesting that the α-diversity had no significant difference among the three groups (Figure 6A). In terms of Figure 6B, PCoA revealed that the gut microbiota was better separated among the three groups in 20-week-old offspring. ANOSIM confirmed the difference between the HF.20 and HFB.20 groups (*R* = 0.6796, *p* = 0.02, data not shown). Figure 6C shows the relative abundance of the top 10 genera. At the genus level, maternal HF significantly reduced the relative abundance of *Lachnoclostridium* (*p* < 0.01), which tends to elevate by maternal betaine (Figure 6D). Consistent with the dams and 4-week-old offspring, the relative abundance of *Romboutsia* was dramatically elevated by maternal betaine feeding compared to HF feeding (*p* < 0.05) (Figure 6E). In Figure 6F, the characteristic gut microbiota is shown in the three groups.

### Altered Gut Microbiota Is Associated With Glucose and Lipid Metabolic Parameters in Dams and Offspring From Young to Adult

**FIGURE 7:**
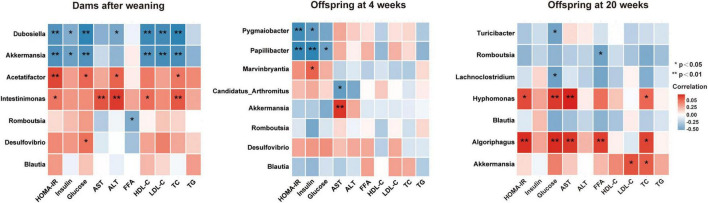
Heatmap of the correlation analysis between the altered genera and glucose and lipid metabolic parameters in dams and offspring. Correlation results in dams after weaning and in offspring at 4 and 20 weeks of age are shown. HOMA-IR, the homeostasis model assessment of insulin resistance; GLU, glucose; AST, aspartate aminotransferase; ALT, alanine transaminase; FFA, free fatty acid; HDL-C, high-density lipoprotein cholesterol; LDL-C, low-density lipoprotein cholesterol; TC, total cholesterol; TG, triglyceride. Values show a significant correlation between the genera and metabolic parameters: *p < 0.05 and **p < 0.01.

To better understand the relationship between gut microbiota and glucose and lipid metabolism, correlation analyses were performed between the altered genera and metabolic parameters in dams and offspring from young to adult (Figure 7). In dams, the *Intestinimonas* and *Acetatifactor*, the two genera significantly reduced by betaine, were positively correlated with HOMA-IR, ALT, and TC (all *p* < 0.05), among which *Intestinimonas* was positively correlated with AST and HDL-C (all *p* < 0.05) and *Acetatifactor* was positively correlated with GLU (*p* < 0.05). Our results showed that *Romboutsia* was negatively correlated with FFAs in dams and 20-week-old offspring. Intriguingly, *Romboutsia* was a genus that was found consistently elevated by maternal betaine in dams and offspring from young to adult. Simultaneously, to the best of our knowledge, this is the first study to find a correlated relationship between *Romboutsia* and altered metabolic parameters in mice.

## Discussion

Adverse nutritional early-life exposure is strongly associated with a great risk for developing obesity and metabolic disorders (Mingrone et al., 2008; Montazeri et al., 2018). While maternal nutrient supplementation during pregnancy could improve perinatal and offspring glucose and lipid metabolism, our previous studies have demonstrated that maternal genistein supplementation (Zhou et al., 2019a,b,c) before and during pregnancy and lactation significantly improved the metabolic disturbances induced by HF in both dams and offspring from young into adulthood, illustrating that nutritional interventions in early life play important roles in reducing the susceptibility of metabolic disorders. Betaine is identified to have low expression in T2DM and GDM populations (Lever et al., 2012, 2014), and diet supplemented with betaine in pregnant mice showed a capability of preventing obesity and diabetes in offspring (Craig, 2004; Ejaz et al., 2016; Chen et al., 2021a). The mechanism behind metabolic benefits of betaine might be due to the regulation of growth and development (Yang et al., 2018), cholesterol catabolism (Zhao et al., 2019), and energy metabolism pathway (Cai et al., 2014, 2016). Increasing number of research articles reported the important roles of gut microbiota in host energy and metabolism hemostasis (Craig, 2004; Ejaz et al., 2016). During pregnancy to early life environment, maternal nutrient and microbiota contributed to the establishment of the infant microbiota and were associated with long-term health (Yao et al., 2021). It certainly merits further exploration whether maternal betaine supplementation influences gut microbiota in both dams and offspring and therefore affects long-term health condition. In the present study, we found that maternal 9-week betaine treatment before and during pregnancy and lactation dramatically improved glucose tolerance, insulin sensitivity, and lipid metabolism induced by HF in dams and offspring from young to adult.

Studies have demonstrated that during pregnancy to early life environment, maternal microbiota have far reaching influence on the establishment of the infant microbiota and associated with long-term health (Yao et al., 2021). There were evidence showing that bran-based diet-induced alteration of gut microbiota was also associated with betaine metabolism, and this result clarified the important role of betaine, as a mediator of the metabolic effect of diet and gut microbiota (Koistinen et al., 2019). Therefore, we hypothesized that gut microbiota may play important roles in explaining the intergenerational metabolic benefits of maternal betaine treatment. Like some natural extracts from daily foods (Sung et al., 2017; Zhou et al., 2019b), our study suggested that betaine supplementation prior to pregnancy and during perinatal period strikingly altered the composition and structure of gut microbiota compared to HF mice, providing evidence supporting that maternal dietary betaine treatment during pregnancy and lactation impacted the establishment of offspring microbiota in the long term.

Indeed, the results showed that betaine significantly reduced the relative abundances of *Acetatifactor* and *Intestinimonas*, which were elevated by HF in dams. *Acetatifactor* grows under anoxic conditions. It has high bile salt hydrolase, 7α-dehydroxylase, and 7β-dehydroxylase activities, which contribute to produce lithocholic acid (LCA) from cholic acid and chenodeoxycholic and ursodeoxycholic acid (Pathak et al., 2018). As a secondary bile acid, LCA is hepatotoxic and plays an important role in liver injury (Fisher et al., 1971; Fickert et al., 2006). Studies have revealed that the administration of LCA to rodents led to intrahepatic cholestasis (Fisher et al., 1971). Fickert et al. (2006) found that administration of LCA for 4 days resulted in hepatocellular necrosis with bile acid absorption disorders in mice, confirming LCA feeding as an experimental model of liver injury. Consistent with these studies, we found that *Acetatifactor* showed adverse effects for metabolism after HF treatment, which was further confirmed by the positive correlation with HOMA-IR, GLU, ALT, and TC. Nevertheless, maternal betaine treatment significantly reduced the unfavorable genus in dams. *Intestinimonas* is a gram-positive anaerobic bacterium. Studies have confirmed that *Intestinimonas* was positively correlated with fecal deoxycholic acid (DCA; Lin et al., 2019), a metabolite known to cause DNA damage (Ridlon et al., 2006). Given that the high level of DCA has been demonstrated to induce obesity-associated hepatocellular carcinoma (Yoshimoto et al., 2013), *Intestinimonas* might have adverse influence on hepatic metabolism. Our research revealed that maternal betaine treatment significantly decreased the relative abundance of *Intestinimonas*, which was positively correlated with the plasma HOMA-IR, AST, ALT, HDL-C, and TC, suggesting that *Intestinimonas* might be associated with the improvement of metabolism in dams.

We also found that maternal betaine supplementation markedly decreased the relative abundance of pathobionts *Desulfovibrio* in 4-week-old offspring. *Desulfovibrio*, as a gram-negative bacterium, can produce hydrogen sulfide and lipopolysaccharide, which was confirmed to be positively related to hyperlipidemia and played a role in mediating gut dysbiosis (Xiao et al., 2014; Wu et al., 2019). Moreover, maternal betaine treatment dramatically enriched the relative abundance of *Blautia* in dams and 4-week-old offspring. *Blautia* is a kind of genus known to contain acetate- and butyrate-producing bacteria and is reported to show a lower relative abundance in T2DM individuals than in healthy controls (Inoue et al., 2017). A case–control study has reported that children with T1DM also showed a lower level of *Blautia* compared to healthy children (Murri et al., 2013). Another randomized clinical trial observed that 12 weeks of metformin treatment significantly increased the level of *Blautia*, which was strongly associated with the beneficial influences in glucose and lipid homeostasis (Tong et al., 2018). These results demonstrated that *Blautia* might be a target in the management of glucose and lipid metabolic disorders in humans. Likewise, *Blautia* were also found to be enriched in rodents with the amelioration of obesity and diabetes (Zhang et al., 2015; Chen et al., 2020). Altogether, maternal betaine treatment alleviated the metabolic disorders induced by maternal HF treatment accompanied by loss of *Desulfovibrio* in 4-week-young offspring and a consistent enrichment of beneficial bacterial *Blautia* both in dams and young offspring. Considering the confirmed beneficial role of *Blautia* in humans, our results provide a rationale for *Blautia* as a target in infants with metabolic abnormalities caused by poor nutritional conditions in early life. As for 20-week-old adult offspring, results demonstrated that maternal betaine observably increased the relative abundance of the genus *Lachnoclostridium*, which was decreased by maternal HF treatment. Recently, *Lachnoclostridium* has been demonstrated to be associated with the mitigation of obesity induced by HF *in vivo* (Wang et al., 2020). Our Spearman’s analysis revealed that *Lachnoclostridium* was negatively correlated with glucose level. Thus, induction of *Lachnoclostridium* might be an ameliorative mechanism of maternal betaine on metabolic disorders in adult offspring, although the impact of *Lachnoclostridium* on glucose and lipid metabolism needs to be further investigated.

Intriguingly, our data suggested that maternal betaine consistently elevated the genus *Romboutsia* compared to the maternal HF group in both dams and offspring from young to adult. The genus of *Romboutsia* is a health indicator in humans (Ricaboni et al., 2016). A study has demonstrated that *Romboutsia* was significantly diminished in obese patients (Martínez-Cuesta et al., 2021). Liu et al. (2018) found that oral hydroxysafflor yellow increased the relative abundances of the genus *Romboutsia*, which might have an influence on its metabolic beneficial effects. Particularly, *Romboutsia sedimentorum* was confirmed to facilitate weight loss for its ability to utilize glucose and produce end products like iso-butanoic and acetic acids (Wang et al., 2015). Therefore, *Romboutsia* might be a critical factor for improving obesity and related glucose and lipid metabolic disturbances. Simultaneously, correlation results showed that *Romboutsia* was negatively correlated with FFAs in dams and 20-week-old offspring. It is the first study to find a correlated relationship between *Romboutsia* and altered metabolic parameters in mice. To sum up, our study proved the consistent enrichment of the genus *Romboutsia* in both dams and offspring from young to adult. To the best of our knowledge, our data provided evidence that maternal betaine supplementation might regulate the *Romboutsia* alteration in offspring from young to adult. Studies demonstrated that *Romboutsia* have been shown to produce SCFAs (Mao et al., 2019; Bojović et al., 2020), which may play important roles in the host health (Zhou et al., 2020). Recent study determined that acetate, propionate, and butyrate, produced by maternal gut microbiota during pregnancy, can be sensed by embryonic intestinal and pancreatic receptors GPR41 and GPR43, which programmed offspring resistance to obesity (Kimura et al., 2020). Consistently, our study highlighted the importance of the SCFA-producing genus *Romboutsia*, which might reveal the potential underlying mechanism in the intergenerational effects of maternal betaine.

In this study, we demonstrated a group of gut microbiota altered in both dams and offspring from young to adult by maternal dietary betaine supplementation, which was associated with HF-induced glucose and lipid metabolic disorders. These results demonstrated that betaine might exert its intergenerational metabolic benefits through regulation of gut microbiota. More importantly, the alteration of *Romboutsia* was discovered in dams and offspring throughout young to adult, which might provide important evidence that neonatal microbiota might be affected by early-life environment and would last lifelong periods. Further microbiota transplantation and metabolite experiments delineating the cause and effect of *Romboutsia* are warranted and also manipulation experiments to deepen the detailed mechanisms from different levels. Overall, the prevention and control window of chronic diseases could be advanced to pregnancy, perinatal, and early-life period.

## Data Availability Statement

The datasets presented in this study can be found in online repositories. The name of the repository and accession number can be found below: National Center for Biotechnology Information (NCBI) BioProject, https://www.ncbi.nlm.nih.gov/bioproject/, PRJNA805394.

## Ethics Statement

All the works were performed according to procedures approved by the Animal Care and Ethics Committee at Peking Union Medical College Hospital (Beijing, China, XHDW-2019-012). All the animal operations were conducted in compliance with the National Institutes of Health guide for the care and use of laboratory animals.

## Author Contributions

JL was responsible for the study design, the animal experiments, and data collection. LD analyzed the data and drafted the manuscript. XZ, DW, CX, XH, and TS helped with the animal experiments and data analysis. MY, QZ, and ML helped with the study design. XX contributed to the whole study design and data interpretation and reviewed the manuscript. All authors approved the final version of this manuscript.

## Conflict of Interest

The authors declare that the research was conducted in the absence of any commercial or financial relationships that could be construed as a potential conflict of interest.

## Publisher’s Note

All claims expressed in this article are solely those of the authors and do not necessarily represent those of their affiliated organizations, or those of the publisher, the editors and the reviewers. Any product that may be evaluated in this article, or claim that may be made by its manufacturer, is not guaranteed or endorsed by the publisher.

## Footnotes

1
http://qiime.org/scripts/split_libraries_fastq.html

2
http://drive5.com/uparse/

3
http://www.arb-silva.de/
