# Supplementary material for: Maternal Dietary Betaine Prevents High-Fat Diet-Induced Metabolic Disorders and Gut Microbiota Alterations in Mouse Dams and Offspring From Young to Adult
Source: Front Microbiol. 2022 Apr 5;13:809642. doi: 10.3389/fmicb.2022.809642 (PMC9037091; doi:10.3389/fmicb.2022.809642)

**Supplementary Material**

**Figure S1. Betaine alters plasma biochemical parameters in high-fat diet dams.**

Dams were analyzed after weaning. (A) TC; (B) TG; (C) LDL-C; (D) HDL-C; (E) ALT (F) AST. Ctr, standard control diet; HFD, high-fat diet; HFB, high-fat diet with betaine. TC, total cholesterol; TG, triglyceride; LDL-C, low-density lipoprotein cholesterol; HDL-C, high-density lipoprotein cholesterol; ALT, alanine transaminase; AST, aspartate aminotransferase. Data are expressed as means ± S.E.M. (n = 5-6/group). One-way ANOVA; *p < 0.05 and **p < 0.01 vs Ctr, #p < 0.05 and ##p < 0.01 vs HFD.


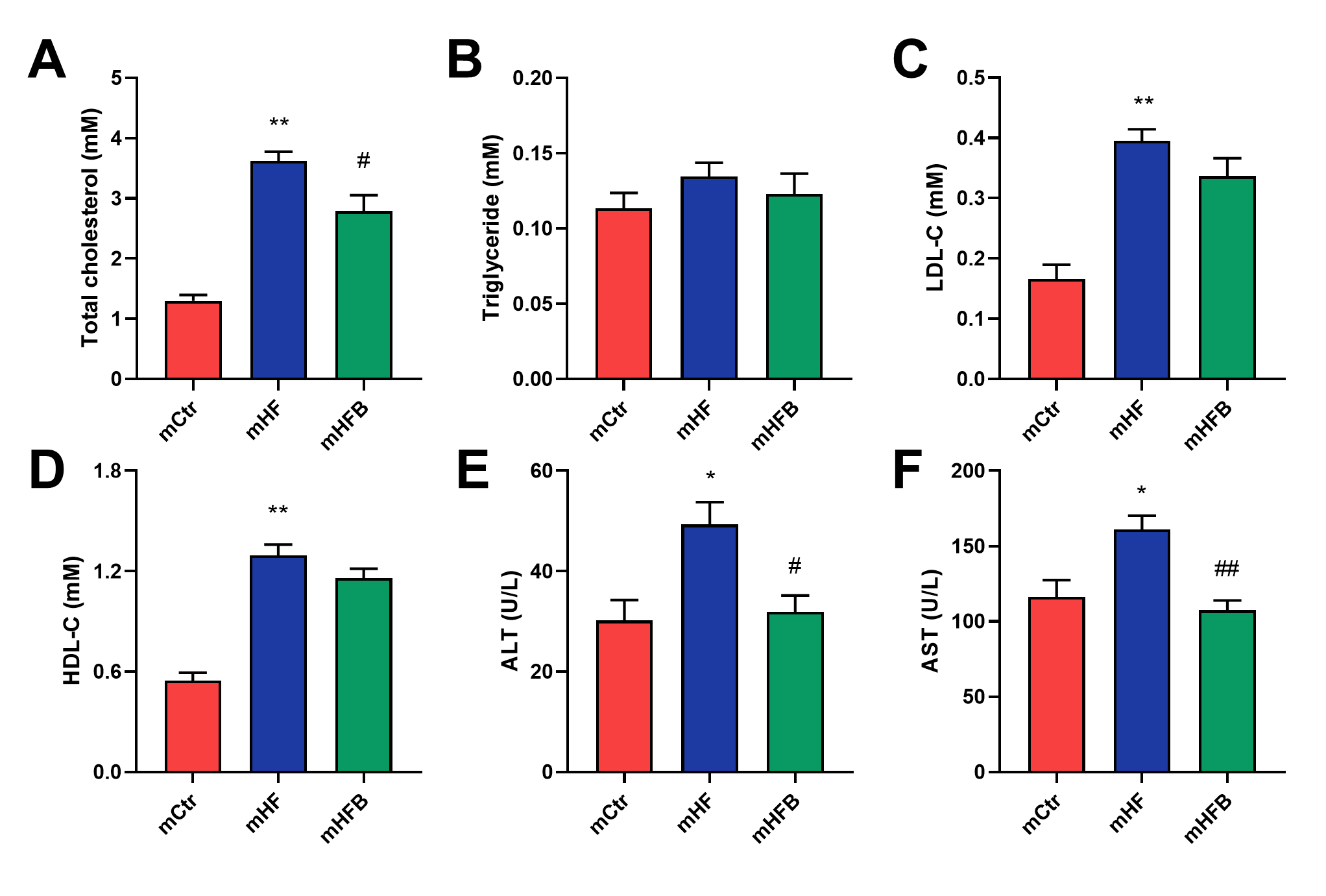


**Figure S2. Maternal betaine alters plasma biochemical parameters in early-life high-fat diet offspring at 4 weeks of age.**

Offspring mice were analyzed at 4 weeks of age. (A) TC; (B) TG; (C) LDL-C; (D) HDL-C; (E) ALT (F) AST. Ctr.4, 4-week offspring of dams fed the standard control diet; HFD.4, 4-week offspring of dams fed the high-fat diet; HFB.4, 4-week offspring of dams fed the high-fat diet with betaine. TC, total cholesterol; TG, triglyceride; LDL-C, low-density lipoprotein cholesterol; HDL-C, high-density lipoprotein cholesterol; ALT, alanine transaminase; AST, aspartate aminotransferase. Data are expressed as means ± S.E.M. (n = 5-6/group). One-way ANOVA; *p < 0.05 and **p < 0.01 vs Ctr, #p < 0.05 and ##p < 0.01 vs HFD.


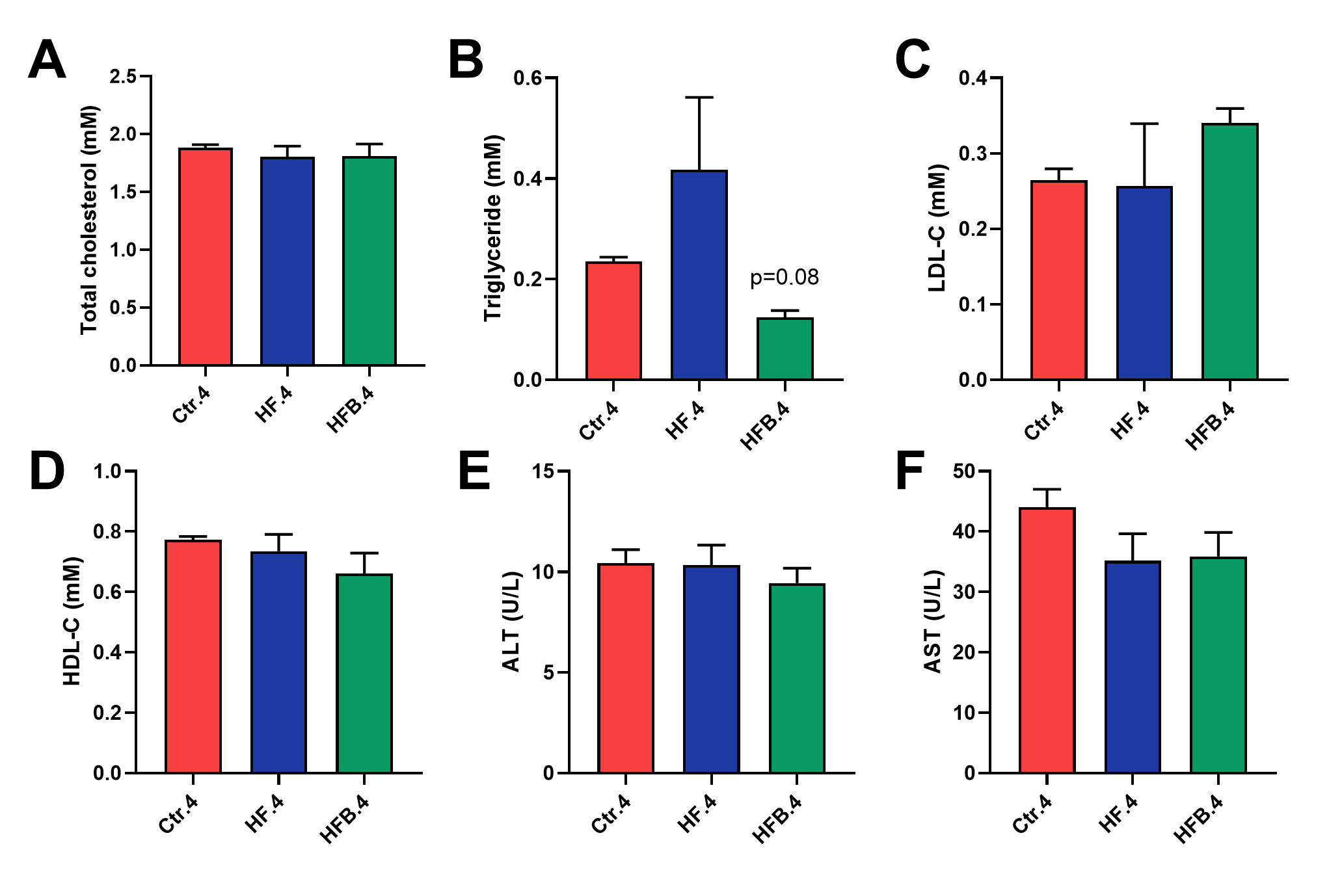


**Figure S3. Maternal betaine alters plasma biochemical parameters in early-life high-fat diet offspring at 20 weeks of age.**

Offspring mice were analyzed at 20 weeks of age. (A) TC; (B) TG; (C) LDL-C; (D) HDL-C; (E) ALT (F) AST. Ctr.20, 20-week offspring of dams fed the standard control diet; HFD.20, 20-week offspring of dams fed the high-fat diet; HFB.20, 20-week offspring of dams fed the high-fat diet with betaine. TC, total cholesterol; TG, triglyceride; LDL-C, low-density lipoprotein cholesterol; HDL-C, high-density lipoprotein cholesterol; ALT, alanine transaminase; AST, aspartate aminotransferase. Data are expressed as means ± S.E.M. (n = 5-6/group). One-way ANOVA; *p < 0.05 and **p < 0.01 vs Ctr, #p < 0.05 and ##p < 0.01 vs HFD.


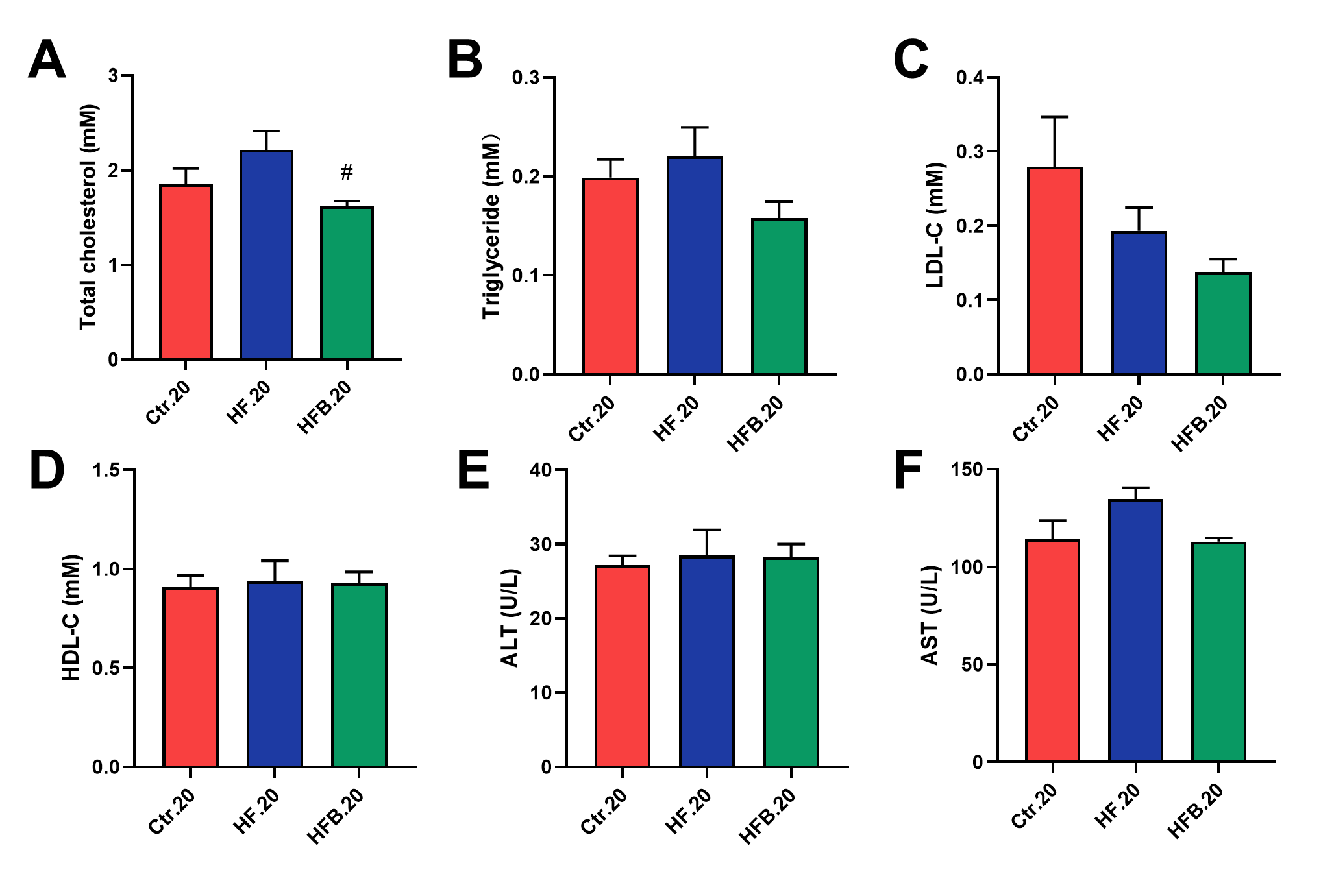

Supplement: Supplementary file 1 [file Data_Sheet_1.docx]
